# Do Global Diversity Patterns of Vertebrates Reflect Those of Monocots?

**DOI:** 10.1371/journal.pone.0056979

**Published:** 2013-05-01

**Authors:** Lynsey McInnes, F. Andrew Jones, C. David L. Orme, Benjamin Sobkowiak, Timothy G. Barraclough, Mark W. Chase, Rafaël Govaerts, Douglas E. Soltis, Pamela S. Soltis, Vincent Savolainen

**Affiliations:** 1 Division of Ecology & Evolution, Imperial College London, Silwood Park Campus, Ascot, United Kingdom; 2 Royal Botanic Gardens, Kew, Richmond, Surrey, United Kingdom; 3 Department of Biology, University of Florida, Gainesville, Florida, United States of America; 4 Florida Museum of Natural History, University of Florida, Gainesville, Florida, United States of America; Consiglio Nazionale delle Ricerche (CNR), Italy

## Abstract

Few studies of global diversity gradients in plants exist, largely because the data are not available for all species involved. Instead, most global studies have focussed on vertebrates, as these taxa have historically been associated with the most complete data. Here, we address this shortfall by first investigating global diversity gradients in monocots, a morphologically and functionally diverse clade representing a quarter of flowering plant diversity, and then assessing congruence between monocot and vertebrate diversity patterns. To do this, we create a new dataset that merges biome-level associations for all monocot genera with country-level associations for almost all ∼70,000 species. We then assess the evidence for direct versus indirect effects of this plant diversity on vertebrate diversity using a combination of linear regression and structural equation modelling (SEM). Finally, we also calculate overlap of diversity hotspots for monocots and each vertebrate taxon. Monocots follow a latitudinal gradient although with pockets of extra-tropical diversity, mirroring patterns in vertebrates. Monocot diversity is positively associated with vertebrate diversity, but the strength of correlation varies depending on the clades being compared. Monocot diversity explains marginal amounts of variance (<10%) after environmental factors have been accounted for. However, correlations remain among model residuals, and SEMs apparently reveal some direct effects of monocot richness. Our results suggest that collinear responses to environmental gradients are behind much of the congruence observed, but that there is some evidence for direct effects of producer diversity on consumer diversity. Much remains to be done before broad-scale diversity gradients among taxa are fully explained. Our dataset of monocot distributions will aid in this endeavour.

## Introduction

Global diversity gradients have so far been studied most extensively in vertebrates with all terrestrial groups showing pronounced latitudinal gradients, i.e., species richness is highest in the tropics (e.g., [1], [2], [3]). Despite an appreciation of the fundamental position of flowering plants as the clade underpinning nearly all terrestrial food webs and providing the structural basis for nearly all terrestrial ecosystems [4], the nature of global diversity gradients in plants remains somewhat elusive. This is due to insufficient use of knowledge of the distribution of the >350,000 estimated angiosperm species. It has led to most studies of diversity patterns in plants being restricted in spatial (e.g., Neotropics: [5]) or taxonomic scope (e.g., palms: [6]), or both (e.g., woody plants in China: [7]).

A recent attempt to study the mechanisms driving the global distribution of vascular plants used species lists taken from regional floras and interpolation techniques to estimate diversity [8], [9]. A strong signal of decreasing richness with increasing latitude was found, further bolstering the generality of the latitudinal diversity gradient [10]. Here, we take a different approach and investigate the global distribution of a major clade within the flowering plants, the monocots (Lilianae sensu [11]; *Monocotyledonae* sensu [12]). Our analysis, focusing on this single, globally-distributed plant clade representing 25% of flowering plant diversity, complements both interpolated and more restricted studies in terms of completeness as we have distribution data for almost all monocot species. Our study has two objectives: (i) to present the global distribution patterns of all monocots; and then (ii) to evaluate their congruence with the distribution patterns of three well-studied vertebrate clades, namely mammals, birds and amphibians. Although vertebrates represent a tiny proportion of the earth's extant species [13], they feature disproportionately in analyses of broad-scale diversity patterns. Therefore, it is important to assess congruence between vertebrates and other taxa, particularly ‘producer species’.

Why monocots? Monocots are a functionally and morphologically diverse group that includes crop plants (e.g., cereals, bananas, pineapples and yams), building material (e.g., bamboos, palms) and ornamentals (e.g., lilies, orchids, irises and daffodils). Monocots are both a useful group with which to evaluate generalities in plant diversity patterns and a sufficiently large and heterogeneous clade that their distribution patterns are also interesting in their own right. They are globally distributed, occupying all terrestrial environments, as well as many aquatic habitats, and are key components of all biomes from tropical forests (e.g., palms, orchids) to tundra (e.g., sedges, grasses) as well as dominating vast areas of some continents (tropical and temperate grasslands).

Explanations for the latitudinal diversity gradient are numerous and span processes that operate on both ecological and evolutionary timescales (e.g., [14]). Abundant and consistent energy input, high resource availability, and greater niche specialisation at low latitudes are all factors thought to contribute to the gradient [15]. Plant diversity can therefore be expected to *directly* influence vertebrate diversity because plants underlie two of the major ecological hypotheses for the latitudinal gradient: as resources for consumption (‘resource diversity’) and as structural elements facilitating niche specialisation (‘vegetation structure’) [16], [17]. Similar diversity gradients in plants and vertebrates may also come about *indirectly* through congruent responses to environmental variables (‘shared environmental effects’) [18].

Jetz *et al.* [17], using the interpolated plant dataset of Kreft & Jetz [9], suggested that the evidence for a direct role of plant diversity driving consumer diversity was limited and that positive correlations in diversity were more likely to be due to similar responses to environmental gradients. Qian & Ricklefs [19] used species lists for 296 geographic units (mostly countries) from the World Resources Institute and found substantial correlations between plant and animal richness, even after controlling for area, environment, topography, and region and attributed this to additional environmental or historical factors that similarly influence both groups. Kissling *et al.* [16], investigating woody plant and bird diversity in Kenya, found evidence in favour of the vegetation structure hypothesis with direct effects of plant diversity on bird diversity even among trophically independent groups. It is clear, therefore, that mechanisms underlying congruent diversity patterns at broad scales remain unresolved with evidence for and against direct effects. In this study, we therefore make a comprehensive attempt to assess the congruence in diversity patterns between a major producer clade (monocots) and three major consumer clades to assess support for direct (‘resource diversity’ or ‘vegetation structure’) or indirect (‘shared environmental effects’) effects of plant diversity on consumer diversity, finding evidence for shared environmental effects alongside support for some direct effects.

## Methods

### Monocot distribution data

The ‘World Checklist of Monocotyledons’ provides a definitive database of all accepted monocot species (∼70,000) and genera and includes distribution data in accord with the third level of the Taxonomic Database Working Group (TDWG) coding system [20] based on herbarium records and expert opinion (further references in [21]). TDWG level 3 (L3) units broadly coincide with countries, but with large countries (USA, Russia, Brazil, Australia, Mexico, China) further subdivided. The median area of L3 units is 130,000 km^2^ but ranges from small islands of 2 km^2^ to large regions ∼ 4 million km^2^ (northern Brazil).

A complete database of the geographic locations of all species of a globally distributed clade represents a significant step forward; however, the discrepancy in size of L3 units compared to the expected range size of individual monocot species remains large, making robust inferences difficult. To overcome this problem, we created a novel database of biome affiliations for 2,647 of the 2,753 monocot genera (96.1% coverage, see omissions below) and merged this with the species-level data to obtain species counts within more narrowly defined units (“L3B units”). We omitted three monocot families from freshwater habitats [Pontederiaceae (6 genera, 33 species), Potamogetonaceae (6 genera, 105 species) and Ruppiaceae (1 genus, 7 species)] because their habitats did not overlap with Olsen *et al.*'s [22] biome classification. We are also missing biome associations for 54 orchid genera (6.2% of all orchid genera) and thus omitted those 322 species. Each L3 unit contains, on average, 2.7 biomes, so by merging the two datasets we more narrowly define presence of individual species into smaller, hopefully more biologically relevant units. Because each unit delimits a relatively homogeneous vegetation type, diversity estimates should more accurately reflect the true diversity of the delimited region. We assumed that each species present in the focal L3 unit is found in all biomes in that unit that have been assigned to its genus. This inevitably inflates the species richness of some L3B units, but still provides a more refined description of diversity patterns (940 units) than relying on L3 units (369 units) or biomes (14 units) alone. We note that each monocot genus occupies on average 1.9 biomes (median  = 1, mean  = 1.9, sd  = 1.4, max  = 13) and that our units do still vary in size between a small tropical dry broadleaf forested island in the Caribbean (∼2 km^2^) to the tropical moist broadleaf forest biome of northern Brazil at >3.5 million km^2^, but the median size is substantially smaller than L3 units at 41,120 km^2^.

To evaluate how our methodology might bias the patterns observed, we also extrapolated species' presences into L3B units in a more conservative way. For those genera that occur in more than one biome within a single L3 unit, we assigned each genus' species (those also assigned to the L3 unit in question) into the set of occupied L3B units in proportion to the size of each unit. As an example, seven species of the orchid genus, *Aa*, are found in the L3 unit, Columbia. The genus is known to occur in both tropical/subtropical grassland and montane grassland biomes, both of which occur in Columbia, although the montane grasslands occupy only one tenth the size of the tropical/subtropical grasslands (15,380 km^2^ versus 152,680 km^2^). In our initial, liberal classification, all seven species were assigned to both biomes. In our conservative classification, one species was assigned to the montane biome (15,380/(15,380+152,680) * 7 = 0.64 and rounded up to 1) and the remaining six species to the lowland biome. In effect, the two methodological approaches bracket the spectrum of possible scenarios: the first, more liberal method, probably overestimates species richness in some cases, whereas the second, more conservative, probably sometimes underestimates species richness.

Monocot species richness within L3B units varies from 0 to 5,425 species with 63 units containing no species (all defined as ice or rock; median  = 400, mean  = 474, sd  = 376). To assess whether particular monocot subclades show greater congruence with vertebrates, we made subsets of the dataset by each order as well as the two most diverse families, Orchidaceae (orchids; 26,128 species) and Poaceae (grasses; 9,485 species). Finally, we also split Orchidaceae into epiphytes (15,062 species) and non-epiphytes (11,066 species), and within Poaceae, C_3_ photosynthetic (5,606 species) and C_4_/CAM photosynthetic (3,879 species) grasses. Note that these last subgroups within Orchidaceae and Poaceae are polyphyletic, representing ecological groups rather than clades. Because of their low diversities, we did not separately assess Acorales (two species) or Petrosaviales (four species).

### Vertebrate distribution data

Vertebrate range polygons were obtained from the Global Amphibian and Mammal Assessments [2], [3] and the AdHoc bird project [1] for all terrestrial amphibian, mammal and bird species (henceforth “vertebrates”). We overlaid the polygons of each taxon onto a projection of our L3B units and extracted their species richness as the number of polygons overlapping each unit. The azonality of the mangrove biome means that these units are typically small and include many spill-over species from neighbouring units. To avoid unwarranted importance being ascribed to mangroves, we removed the 62 L3B units assigned to this biome.

### Correlation analyses

To assess congruence among monocots and vertebrates while accommodating any deviations from normality, we calculated Spearman's Rank correlations between diversities. We avoided the double-zero problem by only including units where there was at least one representative of at least one taxon. Coverage varied between 202 and 814 units occupied by Pandanales and Poales, respectively. Because of the spatial autocorrelation inherent in data of this type, standard significance tests are not appropriate. Instead, we used the Dutilleul *et al.* [23] method to evaluate an effective sample size, taking into account the spatial dependency of the two variables under test.

### Predictive environmental modelling

To evaluate support for direct and indirect effects of monocot diversity on vertebrate diversity, we built two types of statistical model incorporating environmental, regional and landscape predictor variables.

#### 1. Linear regression modelling

First, we built linear models with vertebrate richness as the dependent variable and monocot richness (raw values) as the independent variable. To assess whether observed correlations were a function of similar responses to environmental gradients, we built multiple regression models to explain monocot and vertebrate richness using environmental, landscape and regional (henceforth ‘environmental’) variables. For vertebrates, we also added monocot richness as an additional variable. To control for spatial autocorrelation and associated lack of independence of data points, we built spatially explicit models (error type simultaneous autoregressive models; SAR) with weighted neighbourhood structure (see [24], [25] for more details). We present the non-spatial OLS models for comparison and evaluate the models using AIC and pseudo R^2^.

We also followed Qian & Ricklefs [19] and extracted residuals from each multiple regression model and calculated Spearman's Rank correlations of residuals of each model with residuals from a multiple regression of monocot richness. We acknowledge that there are biases inherent in using residuals as data, namely that fitted models from which residuals are derived do not have identical parameter estimates. However, we use this analysis only as a simple means to assess what correlations remained once environmental effects had been accounted for. Again, we used the Dutilleul *et al.* [23] method to assess significance.

#### 2. Structural equation modelling

Some researchers have argued that multiple regression models are not the most appropriate for evaluating contributions of different factors in explaining variation in a dependent variable, unless each factor has independent effects (e.g., [26]). Because we are assessing the hypothesis that environment similarly affects monocot and vertebrate diversity, we may violate this assumption. In effect, we need a modelling structure that allows for more than one response variable. Structural equation modelling (SEM; [27]) is an alternative technique that can partition correlations between predictor and response variables into direct and indirect effects. SEM can include variables that are both predictor and response variables (here, monocot richness). We first built an *a priori* theoretical model based on established and hypothesised relationships among predictor and response variables, in this case including all environmental variables, monocot and vertebrate richness (separate models for mammals, birds and amphibians). Although it is possible to eliminate paths that are redundant for the most parsimonious explanation of the response variables (e.g., see [16]), we chose to retain the full model structure. This facilitates comparison among models for each vertebrate clade.

### Predictor variables

After preliminary single predictor analyses, we included the following variables in our models: mean annual temperature, temperature seasonality, annual precipitation, precipitation seasonality (all from the Worldclim dataset of average baseline climate data from records between 1960–1990; [28]) and resampled from a resolution of 5 arc-min ( = 0.083°) into our L3B units), elevational range (USGS, [29]), area and region (Africa, Asia-temperate, Asia-tropical, Australasia, Europe, North America or South America). We only include areas above 10,000 km^2^ to avoid spurious importance being assigned to tiny areas; this is the area below which vertebrate distributions are considered unreliable [17], [30]. Diversity patterns on islands are influenced by different processes than mainland regions [31] and are often analysed separately. Removing the smallest areas also removed many islands (98 out of 145 island units). Repeating the analyses without any islands gave qualitatively similar results (data not shown). We used a square-root transformation for precipitation variables and a log10 transformation for all other independent and dependent variables except mean annual temperature.

### Biodiversity hotspots

We identified diversity hotspots for each taxon as those units above the 95% threshold in terms of overall species richness. We assessed hotspot overlap for every pair-wise combination of taxa by calculating the number of hotspots shared by two taxa divided by the total number of hotspots for the taxon with the smaller hotspot set [32]. To test significance, we made 1,000 random hotspot sets for each taxon, and, for each pair-wise combination of taxa, we calculated the proportion of random sets that showed greater overlap than the observed overlap. Finally, for each clade, we calculated the proportion of each of the 13 biomes represented in their hotspot set.

All analyses were conducted in R version 2.13.0 [33].

## Results and Discussion

### Global diversity patterns of monocots

**Figure 1:**
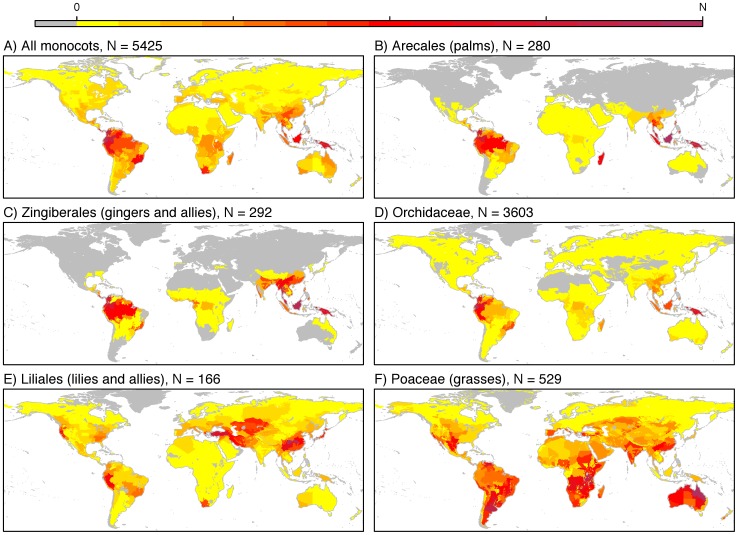
Patterns of monocot diversity. Grey units are unoccupied. (a–f) Untransformed species richness (a) all monocots (b) Arecales (c) Zingiberales (d) Orchidaceae (e) Liliales (f) Poaceae See Fig. S1 for patterns of monocot diversity using the conservative method of assigning species to L3B units. The legend at the top of the figure explains the colour scale used across all maps. The heading for each map gives the richness (N) of the richest unit corresponding to the darkest colour on the colour scale.

**Figure 2:**
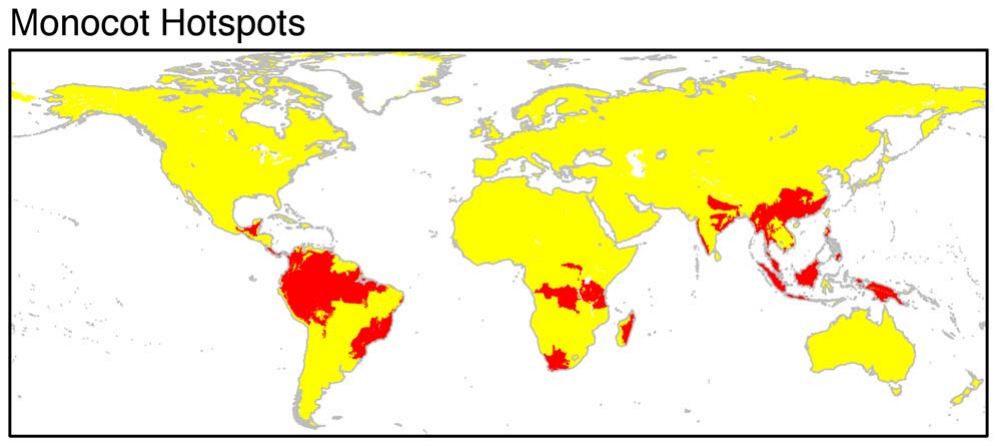
Monocot diversity hotspots. Red units are hotspots (defined as exceeding the 95% threshold in terms of overall species richness).

**Figure 3:**
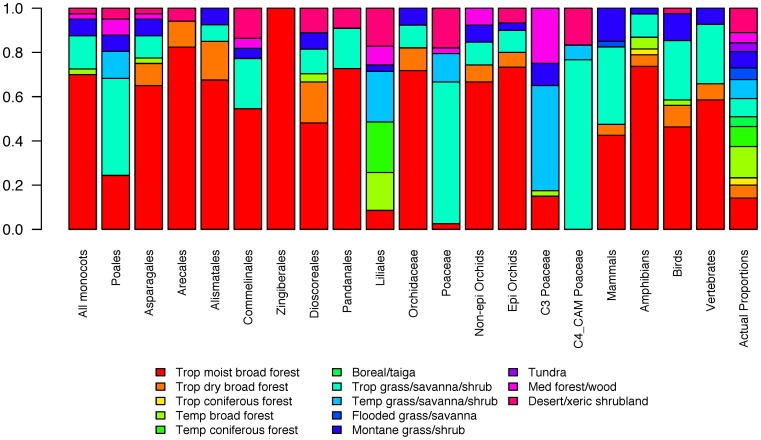
Biome representation in selected hotspots of each taxon. “Actual Proportions” refers to the proportion of all units attributed to each of the 13 biomes. Non-epi/epi Orchidaceae refers to Non-epiphytic/epiphytic orchid species.

Monocots exhibit a strong latitudinal diversity gradient according to both methods of assigning species to L3B units (Figs. 1 & 2, see also Figure S1 for a map of monocot diversity using our conservative method of assigning species to units; Spearman's Rank correlation between the two methods: 0.71). This result is in line with most tests conducted on more restricted taxonomic or regional scales [10]. The Ecuadorian moist tropical forests contain the highest monocot diversity with 5,425 species. This same unit also contains the highest diversity (5,203 species) according to our conservative method. Our simple decomposition of monocots into major subclades also highlighted additional complexity in the structuring of monocot diversity through space. Some subclades are largely restricted to tropical latitudes (e.g., palms, gingers and allies, orchids; Fig. 1b-d). In contrast, others are globally distributed, but with peak diversity at different latitudes: Asparagales in the tropics (largely due to tropical orchid diversity); Liliales at mid-latitudes (Fig. 1e) with diversity s in temperate broadleaf and Mediterranean forests; and grasses (Fig. 1f) with high diversity in tropical, temperate and montane grasslands and Mediterranean-like biomes. Although most monocot diversity is contained within tropical rainforests, substantial diversity is found in other tropical biomes as well as temperate forests and grasslands and, for a number of clades, deserts and other xeric habitats (Fig. 3).

### Correlations between monocot and vertebrate diversity patterns

As expected, we find monocot and vertebrate diversity patterns to be positively associated (Table 1). Correlations using the more conservative methodology of assigning species to L3B units were qualitatively similar (see Figure S1, Table S1) as were correlations calculated using an alternative, parametric coefficient Pearson's *r* (data not shown). Among the three vertebrate clades compared here, amphibian diversity is most strongly associated with monocot diversity (Meng's t test of the difference between dependent correlations using the most conservative effective sample size: t = 3.19, p<0.001 for mammals vs. amphibians and t = 3.49, p<0.001 for birds vs. amphibians). In contrast, the two endothermic clades, mammals and birds, are more strongly associated with each other than with any clade of monocots. Furthermore, although monocots overall show significantly positive correlations with each vertebrate clade, testing each monocot clade separately reveals additional complexity. Clades with peaks of diversity in the tropics exhibit the strongest associations with vertebrates, whereas clades with substantial extra-tropical diversity demonstrate largely independent distributions (Table 1). For example, Poaceae have high extra-tropical diversity as the dominant component of temperate grasslands and desert/xeric shrublands (Fig. 1f). Even focusing exclusively on C_4_ grasses, the major components of tropical grasslands, does not lead to high congruence with any vertebrate group: tropical forest taxa do this much more effectively (Table 1). We also found significant hotspot overlap for monocots with each vertebrate clade, but the proportion of overlap again varied depending on the taxon pair tested (Table 2).

**Table 1:** Cross-taxon congruence of monocots and vertebrates.

Vertebrates	All monocots	Poales	Asparagales	Arecales	Alismatales	Commelinales	Zingi berales	Dio scoreales	Pand anales	Lili ales
Mammals	**0.574**	0.391	**0.609**	0.346	0.413	0.495	0.436	0.484	0.296	0.114?
Amphibians	**0.739**	**0.577**	**0.660**	0.455	**0.707**	**0.594**	**0.540**	**0.697**	0.404	0.089?
Birds	**0.553**	0.381	**0.614**	0.320	0.414	0.438	0.458	0.469	0.301	0.146?

	Orchidaceae	Poaceae	Non-epiphytic Orchids	Epiphytic Orchids	C_3_ Poaceae	C_4/_CAM Poaceae	Amphibians	Birds		
Mammals	**0.514**	0.422	0.459	**0.541**	0.283	0.442	**0.628**	**0.915**		
Amphibians	**0.711**	0.399	**0.674**	**0.696**	0.317	0.371		**0.584**		
Birds	**0.551**	0.386	0.497	**0.559**	0.274	0.397				

All Spearman's rank correlations, apart from those marked with ?, were significant at the 0.05/51 = 0.00098 level according to Dutilleul's test accounting for spatial autocorrelation of neighbouring units and incorporating Bonferroni's correction for multiple tests (n = 51). Correlations above 0.5 are highlighted in bold.

**Table 2:** Hotspot overlap of monocots and monocot subclades with vertebrate taxa.

Vertebrates	All monocots	Poales	Asparagales	Arecales	Alismatales	Commelinales	Zingiberales	Dioscoreales	Pandanales	Liliales
Mammals	0.375	0.375	0.275	0.353	0.350	0.455	0.533	0.296	0.182	0.086?
Amphibians	**0.737**	0.368	**0.605**	**0.824**	**0.658**	0.273	**1.000**	0.481	**0.727**	0.114
Birds	**0.500**	0.463	0.375	0.412	**0.500**	0.364	0.600	0.481	0.273	0.086?
Mammals	40	40	40	17	40	22	15	27	11	35
Amphibians	38	38	38	17	38	22	15	27	11	35
Birds	40	41	40	17	40	22	15	27	11	35

	Orchidaceae	Poaceae	Non-epiphytic Orchids	Epiphytic Orchids	C_3_ Poaceae	C_4/_CAM Poaceae	Amphibians	Birds		
Mammals	0.308	0.231	0.308	0.300	0.075?	0.300	0.395	**0.700**		
Amphibians	**0.632**	0.105	**0.605**	**0.700**	0.158	0.100?		**0.500**		
Birds	0.410	0.256	0.410	0.400	0.125	0.300				
Mammals	39	39	39	30	40	30	38	40		
Amphibians	38	38	38	30	38	30		38		
Birds	39	39	39	30	40	30				

Hotspots were calculated as those units richer than the 95th percentile of the specified diversity measure. Hotspot overlap was calculated as the number of hotspots shared between the two taxa divided by the total number of hotspots in the smaller set (“Denominator”). Overlap above 50% highlighted in bold. All values significant at the 0.05 level except from where indicated.

### Explaining the congruence in diversity patterns

We set out to assess the following hypotheses for why monocot diversity is reflected in vertebrate diversity: 1) directly through vegetation structure or potentially provision of resource diversity; 2) indirectly through shared environmental effects.

If high producer richness leads to high consumer richness directly through the provision of more consumable resources, a high degree of congruence in diversity patterns is expected specifically between producers and primary consumers. Because we were not able to divide our vertebrate dataset into primary and higher-level consumers we are unable to provide a definite conclusion regarding the relevance of the resource diversity hypothesis. We note, however, that through, e.g., food webs and trophic cascades [34], producer diversity could affect higher-level consumers. However, the fact that we find strong associations for mixed groups indicates that provision of diverse resources for consumption is not the primary mechanism by which plant and vertebrate diversities are associated at this scale of analysis, a conclusion shared with a recent analysis that was able to split consumers into primary and higher-level groups [17].

**Figure 4:**
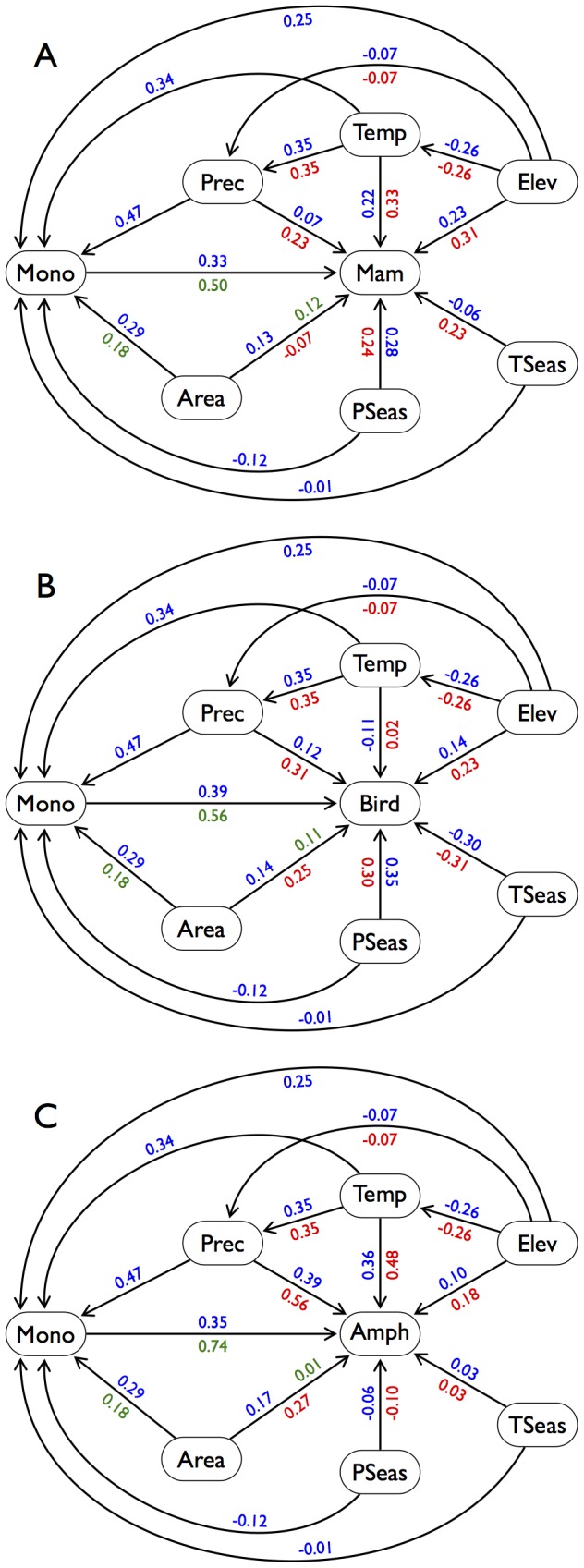
Structural equation models of effects of environmental variables and monocot richness on vertebrate species richness: (a) mammals, (b) birds and (c) amphibians. Values on paths are standardised partial regression coefficients. Because units of our analysis are spatially autocorrelated, significance levels are not given. Green coefficients are from models including only monocot richness and area as predictor variables, red: environmental variables only and blue: environmental variables and monocot richness. Abbreviations: Temp (mean annual temperature), TSeas (temperature seasonality), Prec (annual precipitation), PSeas (precipitation seasonality), Mono (monocot richness).

Dissociating the remaining two hypotheses – vegetation structure versus environment – is confounded by the fact that complex vegetation structure occurs in areas with environmental conditions also expected to accommodate high species diversity [17]. In support of the hypothesis that collinear responses to environmental factors are contributing to the associations observed, once we had accounted for environmental, landscape and regional factors using spatial or non-spatial linear models, we found reduced evidence for a contribution of monocot diversity in driving vertebrate diversity (Table 3, also see Table S2 for estimated model parameters). Furthermore, our SEMs indicated strong direct environmental effects across all taxa (Fig. 4). However, correlations among residuals from multiple regression models remained significantly positive (Table 3), although lower than before accounting for environmental effects (compare with Table 1). Furthermore, our SEM results also indicated direct effects of monocot richness (Fig. 4). These results suggest there may be a direct role for plant richness in facilitating vertebrate diversity, potentially through the provision of physical niche space. However, they could also be due to omission of additional common factors that similarly influence diversification and diversity limits in both monocots and vertebrates, either additional environmental variables or factors relating to the differential overall stability and longevity of habitats within regions [6], [19], [35]. Identifying additional variables that could influence these matching patterns is a research priority.

**Table 3:** Relationships among environment variables, monocot and vertebrate richness.

		OLS			SAR		
		R^2^	AIC	Residual correlations	R^2^	AIC	Residual correlations
Monocots	Environment only	0.509	33.64		0.575	−87.62	
Mammals	Monocots only	0.269	220.98		0.816	−420.87	
	Environment only	0.539	−33.56	0.340	0.849	−498.25	0.278
	Environment + monocots	0.619	−145.87		0.863	−565.38	
Amphibians	Monocots only	0.547	531.23		0.776	189.10	
	Environment only	0.718	269.40	0.486	0.816	68.31	0.359
	Environment + monocots	0.783	113.58		0.839	−23.89	
Birds	Monocots only	0.337	−159.81		0.833	−837.21	
	Environment only	0.546	−364.77	0.396	0.862	−901.49	0.328
	Environment + monocots	0.629	−484.89		0.880	−985.38	

‘Environment’ refers to a multivariate model including the variables: mean annual temperature (°C×10), temperature seasonality (standard deviation of monthly mean temperatures ×100), annual precipitation (mm), precipitation seasonality (coefficient of variation of monthly precipitation), area (km^2^), elevational range (metres) and region (Africa, Asia-temperate, Asia-tropical, Australasia, Europe, North America, South America). Fit is measured using Akaike information criterion (AIC) and pseudo-R^2^. Values in the ‘Residual correlations’ columns refer to the Spearman's Rank correlations between residuals from environmental models of monocot and either mammal, amphibian or bird richness. All correlations are significant at the 0.05/3 = 0.017 level according to the Dutilleul *et al*. [23] method and incorporating Bonferroni's correction (n = 3). Estimated model parameters for all fitted models are in Table S2 in Appendix S2.

### Should we study less inclusive monocot taxa?

We wanted to address whether focussing on less inclusive groups within monocots could be helpful in identifying the mechanisms behind the patterns of congruence observed. For example, Orchidaceae, the largest monocot family, have high tropical diversity (Fig. 1d). Epiphytic orchids grow non-parasitically on other plants or inorganic substrates, derive their nutrients from rain run-off, and are characteristic of tropical rainforests. The distinct microhabitats and fragmented nature of the epiphytic habitat within the forest canopy are thought to contribute to epiphytic species richness [36], but other factors may also be involved such as small size and highly specialised clinging roots [37]. Similarly, almost 5,000 amphibian species depend on moist tropical forests [3]; thus, the high positive association between amphibians and tropical monocots such as epiphytic orchids is likely an indirect relationship stemming from their congruent habitat requirements rather than direct effects (e.g., amphibians are carnivores and many live close to the ground, whereas most orchids occur high in the canopy). Indeed, direct and total environmental effects were most similar between amphibians and monocots (Fig. 4) and, in contrast to the two endothermic taxa, annual precipitation was an important predictor for both amphibians and monocots (Table S2 in Table S2), suggesting that the two taxa might be most closely associated due to shared environmental drivers, their distributions being closely linked to water requirements.

Strong associations may also exist if we considered less-inclusive vertebrates clades or functional groups. For example, grasses and their herbivores and fleshy-fruited plants and their frugivores might be expected to have tightly concordant diversity patterns (e.g., [16], [38]). Here, although we found that mammal richness does peak in the tropical grasslands and savannas of central Africa, in a further analysis, we did not find a strong association between grass (Poaceae) and ungulate (Perissodactyla and Artiodactyla only) diversity patterns (Spearman's rho  = 0.188, p = 0.11), although such an association might be expected given the evidence for co-evolution of grasses and their herbivores [39]. Likewise, Kissling *et al.* [16] did not find stronger associations of frugivores than non-frugivores with fleshy-fruited woody plants in a regional study in Kenya. Although ∼15% of monocot species have fleshy fruits, we were unable to test the fleshy-fruited plant/frugivore hypothesis as we lack global data on the dietary preferences of birds. At small spatial scales, it is well established that the co-evolutionary dynamics of flowering plants and their herbivores lead to positive associations between producer and consumer diversity [40], [41], but it remains unclear whether these local interactions can be generalised to regional scales (see also [17]). Results so far suggest, however, that the strong associations that emerge at our broad scale of analysis are not just the sum of a variety of functionally linked groups such as frugivores with woody plants or herbivores with grasses [16], [38], but are indicative of common drivers structuring the diversity of multiple taxa. Further analyses including additional taxa such as invertebrates should help elucidate the nature of these drivers (e.g., [42]).

### Hotspots and conservation

Conservation planning would be simplified if diverse taxa had congruent diversity hotspots as limited resources could be efficiently targeted to restricted regions of high diversity [43]. In line with previous analyses (e.g., [44]) and our correlation results (Table 1), we find that hotspot overlap rarely reaches above 50% for globally distributed groups. The greatest overlap found was between amphibians and tropical monocot clades with their hotspots centred on the moist tropical broadleaf forest biome (Table 2, Fig. 3). Conservation of the moist tropical broadleaf forest biome therefore appears to be a sound idea based on the distribution of monocots and vertebrates, capturing high numbers of species. On the one hand, it is helpful that hotspots of monocot richness in, for example the Andes and Southeast Asia, coincide with both established hotspots of threatened vertebrate diversity [2], [3], [45] and areas pinpointed by the Sampled Red List Index for Plants (including 1000 monocot species) as particularly threatened (http://threatenedplants.myspecies.info/). However, Sommer *et al.* [46] identified the moist tropical broadleaf forest biome as one expected to lose capacity for plant species richness across a range of climate change scenarios, suggesting that conservation actions in these areas need to incorporate mitigation for species range shifts in order to be effective. Furthermore, monocot subclades with substantial extra-tropical diversity have unique hotspots in other biomes (see also [47]) as does each vertebrate clade [45].

Deciding where to target conservation actions must consider more than just where the greatest numbers of species are. Rather, political and economic feasibility are crucial alongside additional biological considerations such as maintaining intact ecosystems (e.g.,[48]). Our results show that targeting hotspots of single taxa will cover substantial diversity in other taxa, but that there will be a bias towards tropical forest regions. Furthermore, our units are also often larger than is feasible to make protected areas [45]. Nevertheless, inefficiencies in the protected area network have been widely acknowledged and there have been moves to focus conservation on larger areas where viable populations of multiple species can be maintained [49] and where ecosystem services can be maximised [50]. Although we grant that currently our dataset is not ideal for conservation planning, we see scope for this use in the future.

### Limitations and conclusion

All spatial analyses require some consideration of scales of the sampling units.

We believe our units represent a compelling synthesis of an established and robust classification of each monocot species at the country level (political rather than biotic units) with a coarse classification of monocot genera at the biome level (biotically relevant major habitat types, [22]). The correlation between our liberal and conservative methods of assigning monocot species to L3B units was strongly significantly positive and this gives us confidence that our results are robust. We view our methodology as a pragmatic way to increase the resolution of a large amount of valuable species-level distribution data to facilitate further study of this major plant clade and its impact on consumer species. Finally, we advocate further analyses both incorporating additional variables and functional classifications in order to untangle the scale and strength of direct effects of producer diversity on consumer richness.

## Supporting Information

Figure S1 Patterns of monocot diversity using a ‘conservative’ method of assigning species to units. For those genera that occur in more than one biome within a single L3 unit, we assigned each genus' species into the set of L3B units in proportion to the size of each unit (see Methods for more details). White units are unoccupied. Results using the conservative method of assigning species to L3B units.
(DOCX)

Table S1 Cross-taxon congruence of monocots and vertebrates using the conservative method (see Methods) of assigning species to L3B units. All Spearman's rank correlations, apart from those marked with ?, were significant at the 0.05/30 = 0.00167 level according to Dutilleul's test accounting for spatial autocorrelation of neighbouring units and incorporating Bonferroni's correction for multiple tests (n = 30). Correlations above 0.5 are highlighted in bold. Results using the conservative method of assigning species to L3B units.
(DOCX)

Table S2 Estimated parameters for multiple regression models summarised in Table 3. Units <10,000 km^2^ were not included, leaving 601 units in each model. The continent effect is relative to Africa. Precipitation variables were square-root transformed, and all other variables, except mean annual temperature, were log10 transformed. Estimated parameters for multiple regression models.
(DOC)
