# Supplementary material for: Do Global Diversity Patterns of Vertebrates Reflect Those of Monocots?
Source: PLoS One. 2013 May 1;8(5):e56979. doi: 10.1371/journal.pone.0056979 (PMC3641068; doi:10.1371/journal.pone.0056979)

**Fig. S1 - Patterns of monocot diversity using a ‘conservative’ method of assigning species to units.** For those genera that occur in more than one biome within a single L3 unit, we assigned each genus’ species into the set of L3B units in proportion to the size of each unit (see Methods for more details). One units are unoccupied.


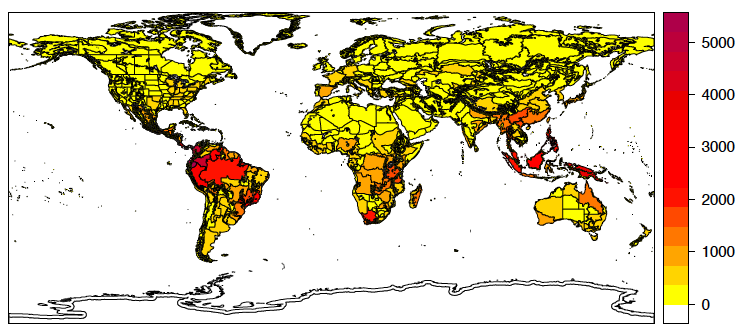

Supplement: Figure S1 — Patterns of monocot diversity using a ‘conservative’ method of assigning species to units. For those genera that occur in more than one biome within a single L3 unit, we assigned each genus' species into the set of L3B units in proportion to the size of each unit (see Methods for more details). White units are unoccupied. Results using the conservative method of assigning species to L3B units. (DOCX) [file pone.0056979.s001.docx]
