# Supplementary material for: Do Global Diversity Patterns of Vertebrates Reflect Those of Monocots?
Source: PLoS One. 2013 May 1;8(5):e56979. doi: 10.1371/journal.pone.0056979 (PMC3641068; doi:10.1371/journal.pone.0056979)
Supplement: Table S1 — Cross-taxon congruence of monocots and vertebrates using the conservative method (see Methods) of assigning species to L3B units. All Spearman's rank correlations, apart from those marked with ?, were significant at the 0.05/30 = 0.00167 level according to Dutilleul's test accounting for spatial autocorrelation of neighbouring units and incorporating Bonferroni's correction for multiple tests (n = 30). Correlations above 0.5 are highlighted in bold. Results using the conservative method of assigning species to L3B units. (DOCX) [file pone.0056979.s002.docx]

**Table S1** - Cross-taxon congruence of monocots and vertebrates using the conservative method (see Methods) of assigning species to L3B units. All Spearman’s rank correlations, apart from those marked with ^, were significant at the 0.05/30 = 0.00167 level according to Dutilleul’s test accounting for spatial autocorrelation of neighbouring units and incorporating Bonferroni’s correction for multiple tests (n = 30). Correlations above 0.5 are highlighted in bold.
