# Supplementary material for: Do Global Diversity Patterns of Vertebrates Reflect Those of Monocots?
Source: PLoS One. 2013 May 1;8(5):e56979. doi: 10.1371/journal.pone.0056979 (PMC3641068; doi:10.1371/journal.pone.0056979)
Supplement: Table S2 — Estimated parameters for multiple regression models summarised in Table 3. Units <10,000 km2 were not included, leaving 601 units in each model. The continent effect is relative to Africa. Precipitation variables were square-root transformed, and all other variables, except mean annual temperature, were log10 transformed. Estimated parameters for multiple regression models. (DOC) [file pone.0056979.s003.doc]

**Table S2** – Estimated model parameters for ‘environmental’ models

**Table S2 continued**
